# Supplementary material for: Elastic Fibers and F-Box and WD-40 Domain-Containing Protein 2 in Bovine Periosteum and Blood Vessels
Source: Biomimetics (Basel). 2022 Dec 23;8(1):7. doi: 10.3390/biomimetics8010007 (PMC9844355; doi:10.3390/biomimetics8010007)
Supplement: Supplementary file 1 [file biomimetics-08-00007-s001.zip › biomimetics-2024284-supplementary.pdf]

Supplementary Material

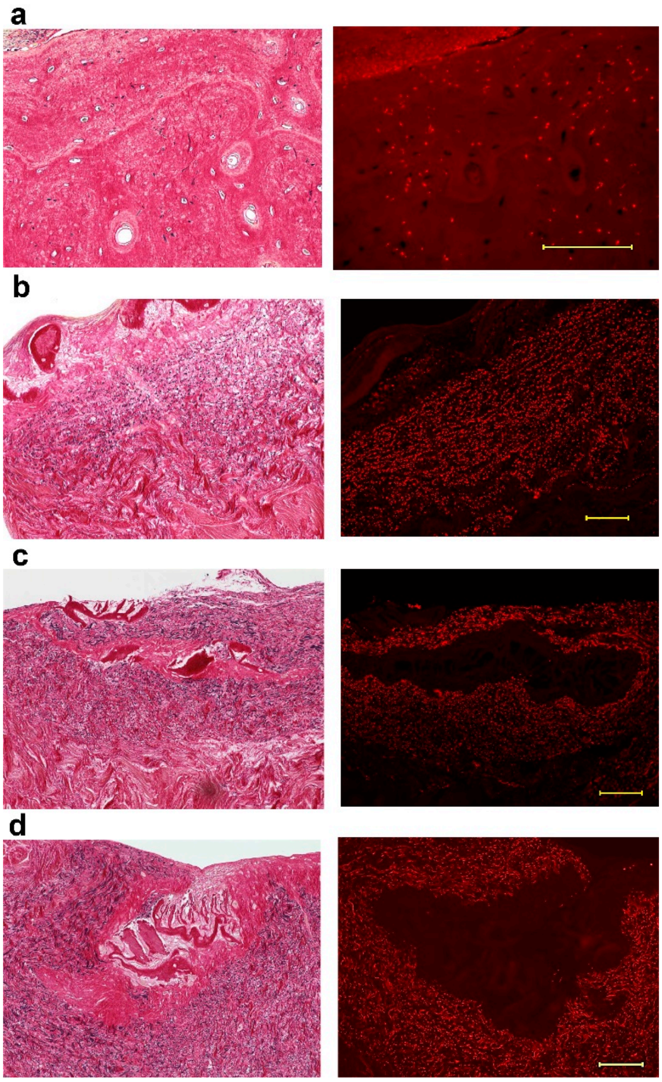

Figure S1. Comparison of (left) Elastica van Gieson (EVG) staining. and (right) immunostaining of FBXW2 (red). (a) Bone. (b)-(d) Periosteum during explant culture. (b) 3 weeks (c) 4 weeks (d) 5 weeks. Scale bar: 100 μm.

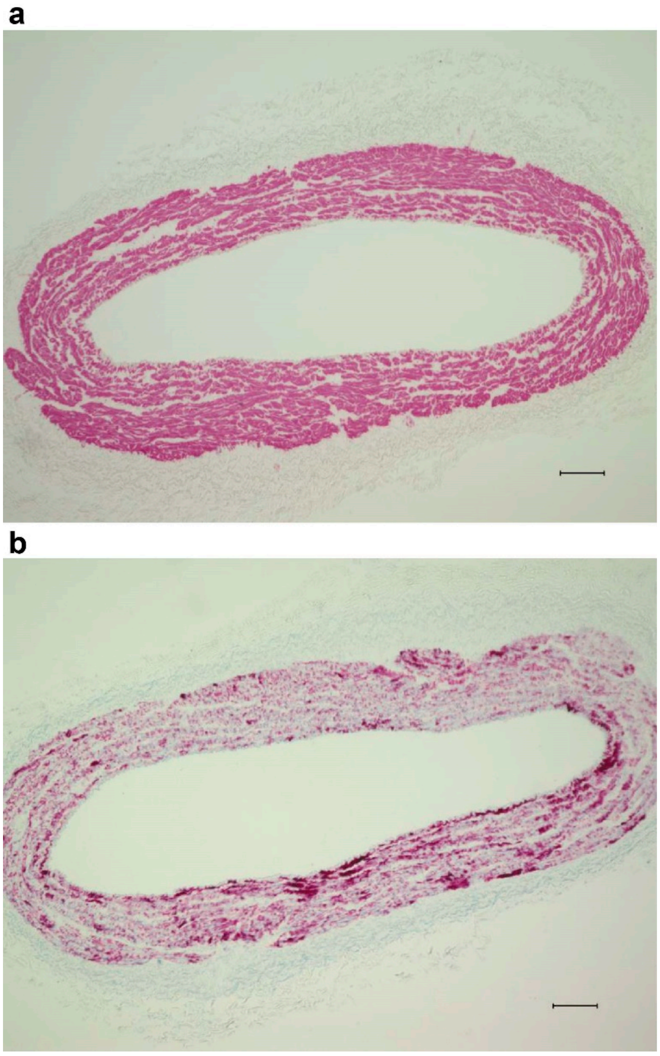

Figure S2. Comparison of single immunostaining and double immunostaining. Observation of large blood vessels. (a) Immunostaining of blood vessels with alkaline phosphatase (AP) tag. αSMA: red. (b) Immunostaining of blood vessels with AP tag. Osteocalcin: blue, αSMA: red. Scale bar: 100 μm..

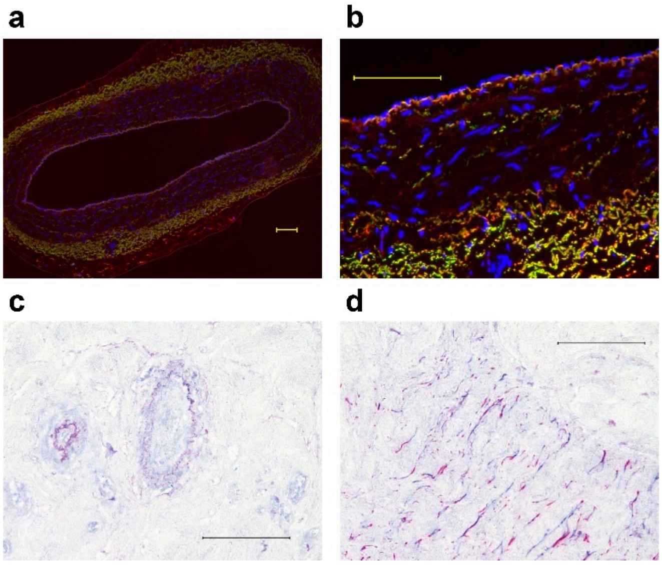

Figure S3. Double immunostaining of elastin and FBXW2. (a), (b) Large blood vessels. Elastin: green. FBXW2: red. (a) low magnification (b) high magnification of (a). (c), (d) Periosteum during explant culture (3 weeks). Elastin: bule. FBXW2: red. Scale bar: 100 μm..
